# Long-term continuous degradation of carbon nanotubes by a bacteria-driven Fenton reaction

**DOI:** 10.3389/fmicb.2023.1298323

**Published:** 2023-11-30

**Authors:** Seira Takahashi, Katsutoshi Hori

**Affiliations:** Department of Biomolecular Engineering, Graduate School of Engineering, Nagoya University, Nagoya, Aichi, Japan

**Keywords:** carbon nanotube, degradation, Fenton reaction, bacteria, *Shewanella*

## Abstract

Very few bacteria are known that can degrade carbon nanotubes (CNTs), and the only known degradation mechanism is a Fenton reaction driven by *Labrys* sp. WJW with siderophores, which only occurs under iron-deficient conditions. No useful information is available on the degradation rates or long-term stability and continuity of the degradation reaction although several months or more are needed for CNT degradation. In this study, we investigated long-term continuous degradation of oxidized (carboxylated) single-walled CNTs (O-SWCNTs) using bacteria of the genus *Shewanella*. These bacteria are widely present in the environment and can drive the Fenton reaction by alternating anaerobic-aerobic growth conditions under more general environmental conditions. We first examined the effect of O-SWCNTs on the growth of *S. oneidensis* MR-1, and it was revealed that O-SWCNTs promote growth up to 30 μg/mL but inhibit growth at 40 μg/mL and above. Then, *S. oneidensis* MR-1 was subjected to incubation cycles consisting of 21-h anaerobic and 3-h aerobic periods in the presence of 30 μg/mL O-SWCNTs and 10 mM Fe(III) citrate. We determined key factors that help prolong the bacteria-driven Fenton reaction and finally achieved long-term continuous degradation of O-SWCNTs over 90 d. By maintaining a near neutral pH and replenishing Fe(III) citrate at 60 d, a degraded fraction of 56.3% was reached. *S. oneidensis* MR-1 produces Fe(II) from Fe(III) citrate, a final electron acceptor for anaerobic respiration during the anaerobic period. Then, ·OH is generated through the Fenton reaction by Fe(II) and H_2_O_2_ produced by MR-1 during the aerobic period. ·OH was responsible for O-SWCNT degradation, which was inhibited by scavengers of H_2_O_2_ and ·OH. Raman spectroscopy and X-ray photoelectron spectroscopy showed that the graphitic structure in O-SWCNTs was oxidized, and electron microscopy showed that long CNT fibers initially aggregated and became short and isolated during degradation. Since *Shewanella* spp. and iron are ubiquitous in the environment, this study suggests that a Fenton reaction driven by this genus is applicable to the degradation of CNTs under a wide range of conditions and will help researchers develop novel methods for waste treatment and environmental bioremediation against CNTs.

## 1 Introduction

Carbon nanotubes (CNTs), especially single-walled CNTs (SWCNTs), have a wide range of applications due to their excellent properties, such as mechanical strength, optical properties, and electrical and thermal conductivity (Dresselhaus et al., 2004; Byrne et al., 2018; Hu et al., 2023). However, there are concerns about their impact on human health and ecosystems (Poland et al., 2008). Some CNTs are needle-like, similar to asbestos, and can induce mesothelioma, pleural fibrosis, and lung cancer (Donaldson et al., 2013). Recent studies have reported that some CNTs are also toxic to plants, animals, and microorganisms, and may alter biodiversity (Chen et al., 2017; Mendonca et al., 2017; Chen et al., 2018; Kong et al., 2023); Such CNTs cause growth inhibition and reduced seed germination in plants (Begum and Fugetsu, 2012; Hatami, 2017), embryo growth inhibition and pneumonia in animals (Roman et al., 2013; Al Moustafa et al., 2016; Fujita et al., 2016), and cell membrane damage in microorganisms (Liu et al., 2009; Yadav et al., 2016). As the use of CNTs has become more widespread, the amount of CNTs released into the environment, either accidentally or as waste, may also increase, leading to additional concerns. Therefore, the safety against human health and biodegradability of CNTs have attracted great attention.

Allen et al. (2008, 2009) first reported the degradation of oxidized (carboxylated) SWCNTs (O-SWCNTs), which are functionalized SWCNTs, by horseradish peroxidase (HRP). Since then, the biodegradation of CNTs by incubation with heme enzymes, such as human myeloperoxidase (Kagan et al., 2010), human eosinophil peroxidase (Andon et al., 2013), bovine lactoperoxidase (Bhattacharya et al., 2015), fungal manganese peroxidase (Zhang et al., 2014) and lignin peroxidase (Chandrasekaran et al., 2014), in the presence of their substrate (H_2_O_2_) has been reported. However, Flores-Cervantes et al. (2014) were the first to quantitatively investigate the enzymatic degradation of CNTs and estimated a half-life of 80 years for SWCNT degradation by HRP. This result was largely different from the rapid degradation described in the paper by Allen et al. (2009), in which CNTs disappeared within 10 d. Quantitative studies and reproducibility have not yet been conducted for enzymes other than HRP. In addition, we recently demonstrated that the degradation of CNTs during incubation with heme enzymes and externally added H_2_O_2_ is not caused by enzymatic reaction but rather the Fenton reaction, which produces hydroxyl radicals (·OH) that are highly reactive and can oxidize most organic substances rapidly and nonselectively, via the decomposition of H_2_O_2_ catalyzed by Fe(II) (Qin et al., 2015; Takahashi et al., 2023). Peroxidases are easily inactivated by their substrate H_2_O_2_ via heme degradation, which is called suicide inactivation (Valderrama et al., 2002). In our previous study, the rapid suicide inactivation of peroxidases, including HRP, was observed; this process depended on the H_2_O_2_ concentration and was accompanied by the release of iron, which caused the Fenton reaction (Takahashi et al., 2023). Thus, enzymatic degradation of CNTs may not be as promising as previously expected.

Bacteria have an immense and diverse metabolic system and are utilized for environmental bioremediation and waste treatments. For CNTs, biotransformation or biodegradation by *Trabusiella guamensis* (Chouhan et al., 2016), *Mycobacterium vanbaalenii* PYR-1 (You et al., 2017), and a bacterial community consisting of *Burkholderia kururiensis*, *Delftia acidvorans* and *Stenotrophomonas maltophilia* (Zhang et al., 2013) has also been reported. However, the molecular mechanisms underlying these bacterial transformations of CNTs have never been elucidated. Wang et al. (2020) showed that *Labrys* sp. WJW secretes siderophores under iron-deficient conditions and degrades CNTs as the sole carbon source; this process occurs through an extracellular biogenic Fenton-like reaction induced by Fe(II) that is generated through the reduction of Fe(III) by siderophores and autocrine H_2_O_2_. This is the first and only report in which the mechanism underlying the degradation of carbon nanomaterials by bacteria was shown, suggesting that the Fenton reaction induced by microorganisms by different pathways may contribute to the degradation of CNTs.

*Shewanella* spp., environmentally ubiquitous facultative anaerobic bacteria, produce Fe(II) by reducing Fe(III) under anaerobic conditions and H_2_O_2_ by reducing O_2_ under aerobic conditions, thus efficiently inducing the Fenton reaction by alternating anaerobic-aerobic cultures. The degradation of pentachlorophenol (McKinzi and Dichristina, 1999), 1,4-dioxane (Sekar and DiChristina, 2014), enrofloxacin (Yan et al., 2016), polybrominated diphenyl ethers (Peng et al., 2020; Shi et al., 2021), and polystyrene (Yang et al., 2022) by the Fenton reaction driven by *Shewanella* spp. has been reported. Therefore, the method should be effective in the degradation of CNTs, albeit much more recalcitrant than the other chemicals. The Fenton reaction driven by *Shewanella* spp., such as *S. oneidensis* and *S. putrefaciens*, could be applied to the degradation of CNTs under a wide range of environments, not limited to iron-deficient conditions. However, it is unclear whether *Shewanella* spp. is resistant to CNTs and whether the bacteria-driven Fenton reaction continues long enough to degrade CNTs. The purpose of this study is to determine the effectiveness of the Fenton reaction driven by *Shewanella* spp. for the continuous degradation of CNTs.

## 2 Materials and methods

### 2.1 Culture medium and chemical reagents

Dispersions of O-SWCNTs (Product No. ZEONANOR-SG101) were produced and provided by Zeon Nanotechnology Co., Ltd. (Japan). The dispersions of O-SWCNTs were stored in the dark, and it was confirmed that the properties of the pristine O-SWCNTs were unchanged from the beginning to the end of the experiment by Raman spectroscopy. Luria–Bertani (LB) broth and DL-sodium lactate were purchased from Nacalai Tesque Co., Ltd. (Japan). Mannitol and catalase were purchased from FUJIFILM Wako Pure Chemical Industries Co., Ltd. (Japan). Fe(III) citrate and horseradish peroxidase were purchased from Sigma–Aldrich, Ltd. (USA). 3′-(p-hydroxyphenyl) fluorescein (HPF) was purchased from Goryo Chemical, Inc. (Japan). N,N-diethyl-1.4-phenykenediamine sulfate (DPD) was purchased from Tokyo Chemical Industry Co., Ltd. (Japan). Ferrozine (3-(2-pyridyl)-5,6-bis (4-sulfophenyl)-1,2,4-triazine) was purchased from Dojindo Laboratories, Ltd. (Japan). N_2_ gas (99.99% purity) was purchased from Alpha System Co. (Japan).

### 2.2 Evaluation of the effect of O-SWCNTs on *S. oneidensis* MR-1 growth

*S. oneidensis* MR-1 was precultured aerobically in LB medium until the optical density at 600 nm (OD_600_) reached 1.0 and harvested by centrifugation. The cells were resuspended in M1 medium containing 20 mM sodium lactate (Peng et al., 2020; Supplementary Table S1) to an OD_600_ of 0.1 after they were washed three times with the medium and grown aerobically for 24 h at 30°C in 25 mL of M1 medium with different concentrations of O-SWCNTs in 100-mL flasks. The culture broth was periodically collected and diluted stepwise in M1 medium, of which 100 μL was spread on LB agar plates. After 48 h of incubation at 30°C, the number of colonies was counted to determine the colony forming units (CFUs).

### 2.3 Degradation of O-SWCNTs by *S. oneidensis* MR-1

A suspension of *S. oneidensis* MR-1 cells in 25 mL of M1 medium containing 20 mM sodium lactate was prepared in a flask as described above. Fe(III) citrate and O-SWCNTs were added to the cell suspension at final concentrations of 10 mM and 30 μg/mL, respectively, and N_2_ gas was bubbled from a gas exchange tube connected to a silicone plug capping the flask for 10 min. After the gas exchange tube was clamped, cells were incubated anaerobically in the flask at 30°C for 21 h. The culture was then continued aerobically and air was bubbled from the gas exchange tube for 3 h at 30°C. This 24 h cycle consisting of 21-h anaerobic and 3-h aerobic incubations was repeated 90 times (90 d) in the dark. Sodium lactate and/or lactic acid were supplied manually using a pipette daily as an electron donor at a final concentration of 10 mM at the end of the aerobic incubation. The approximate pH of the culture medium during incubation was checked using pH test strips at the end of the aerobic incubation. If intended, the pH was returned to approximately 7 daily using sodium lactate when the pH was below 8 and lactic acid and/or sodium lactate when the pH was above 8. For a control experiment, Fe(III) citrate was replaced by nitrate (10 mM). For other control experiments, 100 ng/mL catalase was supplied daily as an H_2_O_2_ scavenger, or 240 mM mannitol was initially added to M1 medium as a ·OH scavenger. The concentration of Fe(II) was measured at the end of each anaerobic and aerobic incubation with a ferrozine-based detection method (Dichristina, 1992; Yang et al., 2022). The concentration of H_2_O_2_ was measured at the end of each aerobic incubation using a method modified for iron-containing samples by Katsoyiannis et al. (2008) and Peng et al. (2020), based on peroxidase-catalyzed oxidation of DPD. The concentration of ·OH in the supernatant was measured 1.5 h after the start of each aerobic incubation using a fluorescent probe (HPF, 5 μmol/L) (Hessler et al., 2012; Wang et al., 2020).

### 2.4 Quantification and characterization of O-SWCNTs during and after incubation

To the whole culture broth sample (25 mL), 500 μL of 10 mg/mL lysozyme solution (in Tris–HCl buffer, pH 8) was added, and after the solution was stirred for 30 min, an equal volume of 5% SDS solution was added, and the broth was heated at 60°C for 2 h for cell lysis. The precipitate containing O-SWCNTs was collected by ultracentrifugation (35,000 rpm, 15 min, 20°C), rinsed with ultrapure water (resistivity at 25°C, >18 MΩ cm; TOC, <5 ppb), washed with 3 M HCl to remove iron oxides formed during incubation, rinsed with ethanol, and redispersed in ultrapure water. In every rinsing and washing process above, the pellet was redispersed and recovered by ultracentrifugation three times. The cellular components that could not be removed by rinsing and washing were sedimented by centrifugation (3,000 rpm, 2 min, 25°C). O-SWCNTs were recovered from the supernatant by subsequent ultracentrifugation (35,000 rpm, 15 min, 20°C) and redispersed in 25 mL of 5% SDS solution by sonication for 10 min. Our previous work confirmed that the properties of the O-SWCNTs used in this study are not affected by sonication (Takahashi et al., 2023). The absorbance of the resulting O-SWCNT dispersion at 750 nm (A_750_) was measured to calculate the concentration of O-SWCNTs from a calibration curve prepared using stocks of O-SWCNT dispersion.

Prior to instrumental analysis, O-SWCNTs were collected again by ultracentrifugation under the same conditions above, washed with ultrapure water, and redispersed in methanol by sonication for 10 min. Specimens for Raman spectroscopy were prepared by drop-casting approximately 50 μL of the suspension on glass microscope slides and drying. All spectra were collected on a micro-Raman spectrometer (inVia Reflex, Renishaw, UK) using an excitation wavelength of 532 nm. The samples were scanned from 1,000–1,900 cm^−1^ to visualize the D and G bands of O-SWCNTs. Spectra were collected with a 10 s exposure time and averaged across 5 scans per sample. In each sample, at least 10 different spots were analyzed. For X-ray photoelectron spectroscopy (XPS) (ESCALAB250, VG Scientific, UK), specimens were prepared by drop-casting approximately 100 μL of the samples on carbon tape and drying. XPS was performed using monochromatized Al Kα radiation (*hv* = 1486.6 eV) as an X-ray source. For transmission electron microscopy (TEM), 10 μL of the appropriately diluted suspensions were dropped onto a carbon-coated copper grid (ELS-C10, Okenshoji Co., Ltd., Tokyo, Japan) and dried overnight under ambient conditions prior to TEM imaging. TEM images were acquired using a JEM-2100Plus (JEOL, Ltd., Tokyo, Japan) equipped with a CCD camera and operated at an acceleration voltage of 200 kV.

## 3 Results

### 3.1 Incubation conditions for the continuous Fenton reaction by *S. oneidensis* MR-1 in the presence of O-SWCNTs

**Figure 1:**
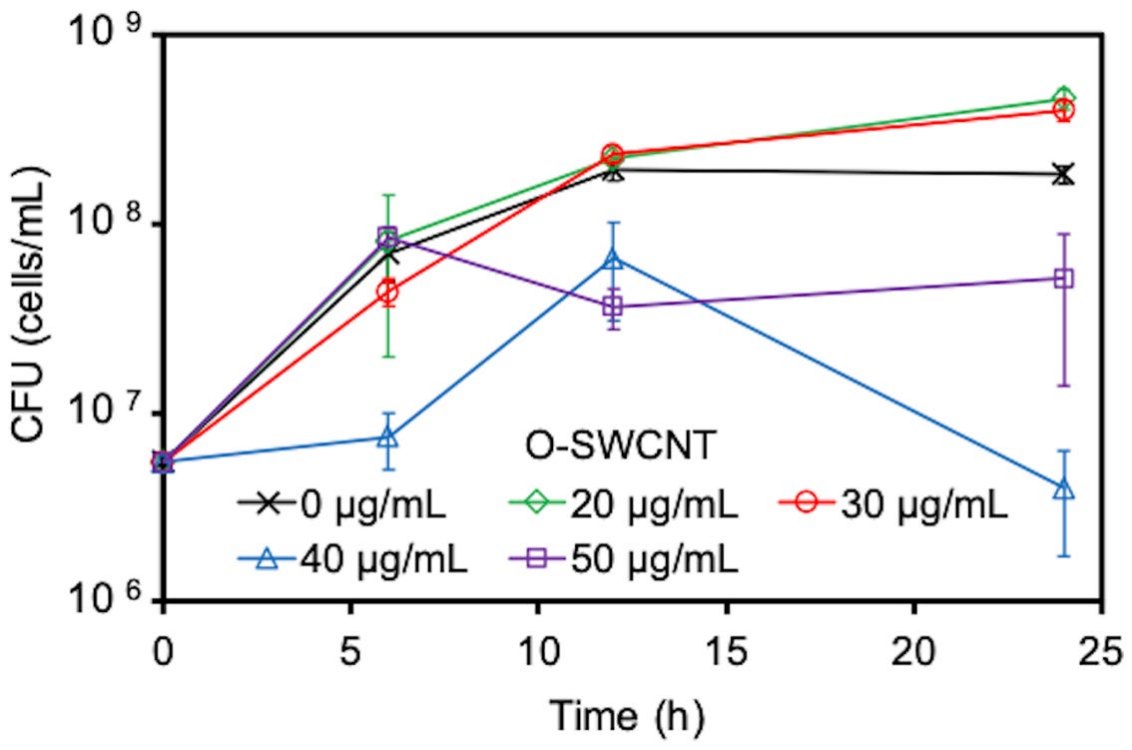
Effect caused by the concentration of O-SWCNTs on *S. oneidensis* MR-1 growth. Error bars indicate standard error from three independent cultures.

Due to concerns that the cytotoxicity of O-SWCNTs would kill or inactivate bacterial cells and rapidly stop the bacteria-driven Fenton reaction, we first examined the effect of O-SWCNTs on the growth of *S. oneidensis* MR-1. For this purpose, cells were grown aerobically in the presence of different concentrations of O-SWCNTs at 30°C for 24 h, and the change in CFUs with incubation time was analyzed. Despite the reported toxicity of CNTs, the addition of 20 and 30 μg/mL O-SWCNTs unexpectedly promoted the growth of *S. oneidensis* MR-1, which was greater than that without O-SWCNTs (Figure 1 and Supplementary Figure S1). However, the growth was greatly inhibited at 40 μg/mL or greater. Accordingly, subsequent incubations of *S. oneidensis* MR-1 with O-SWCNTs in this study were conducted at an O-SWCNT concentration of 30 μg/mL.

**Figure 2:**
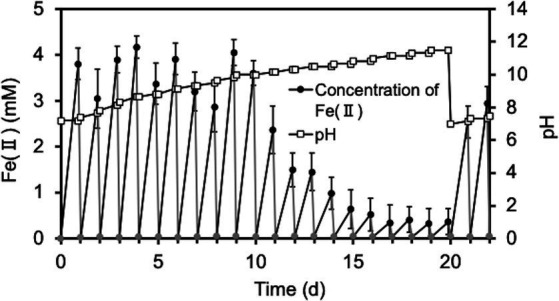
Time courses of pH and the Fe(II) concentration during the cultivation of *S. oneidensis* MR-1 with 24-h incubation cycles consisting of 21-h anaerobic and 3-h aerobic periods in the presence of O-SWCNTs and without controlling pH. The pH was adjusted to 7 at the end of the 20-d incubation. The square symbols indicate the pH values. Circular symbols indicate Fe(II) concentrations during the anaerobic (black) and aerobic (gray) periods. Error bars indicate standard error from three independent incubations.

**Figure 3:**
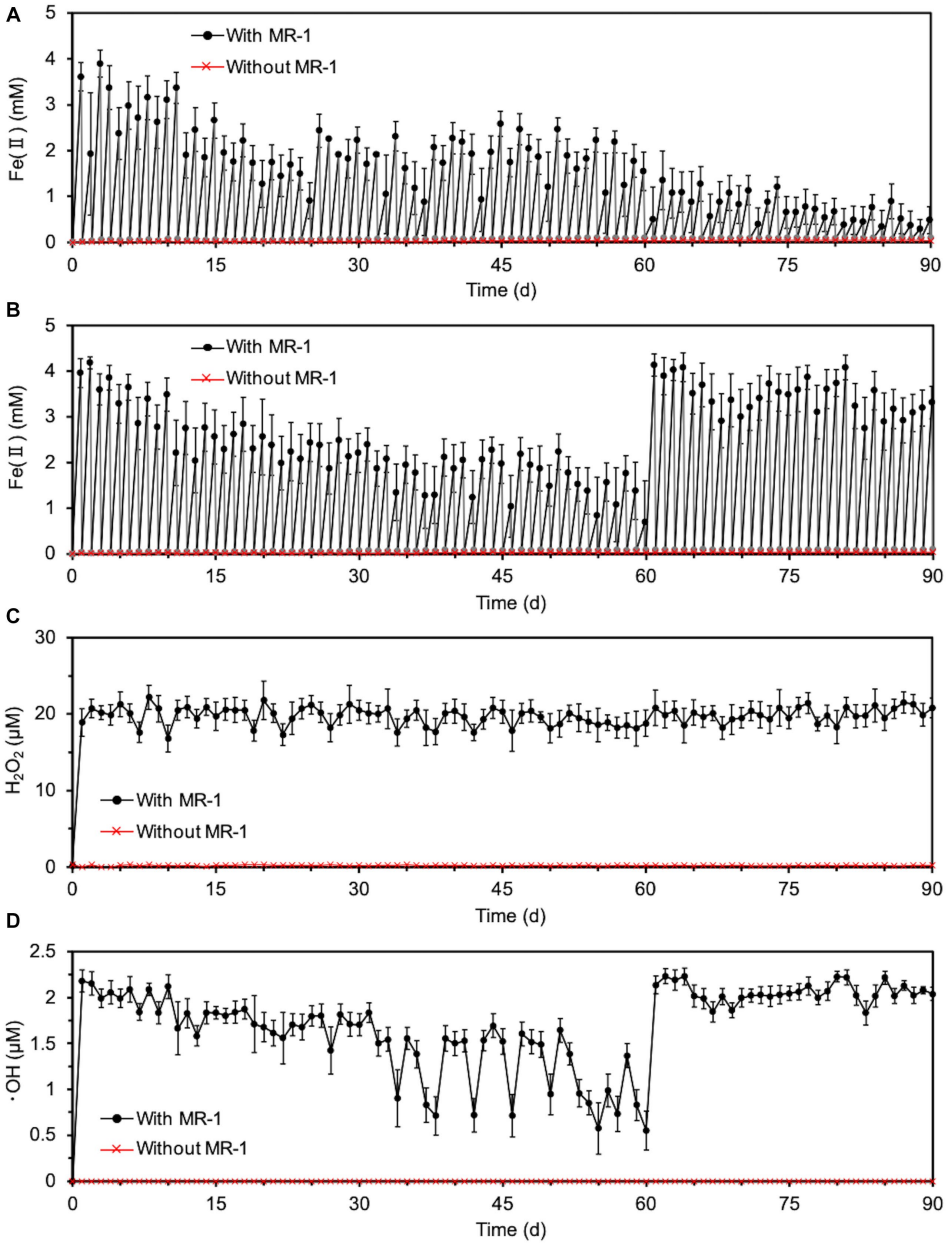
Long-term Fenton reaction driven by *S. oneidensis* MR-1 in 24-h incubation cycles consisting of 21-h anaerobic and 3-h aerobic periods under daily controlled pH near neutral in the presence of O-SWCNTs. Error bars indicate standard error from three independent incubations. **(A)** The time course of Fe(II) concentrations during the anaerobic (black) and aerobic (gray) periods without the replenishment of Fe(III) citrate. In the data without MR-1 (red), only the concentration of Fe(II) at the end of each anaerobic period was shown to simplify the figure. The concentration of Fe(II) was also almost zero at the end of each aerobic period, although the data were not shown. **(B)** The time course of Fe(II) concentrations during the anaerobic (black) and aerobic (gray) periods with the replenishment of Fe(III) citrate at 60 d. In the data without MR-1 (red), only the concentration of Fe(II) at the end of each anaerobic period was shown to simplify the figure. The concentration of Fe(II) was also almost zero at the end of each aerobic period, although the data were not shown. **(C)** The time course of H_2_O_2_ concentrations during alternating anaerobic-aerobic incubations of *S. oneidensis* MR-1 with the replenishment of Fe(III) citrate at 60 d. **(D)** The time course of ·OH concentrations during alternating anaerobic-aerobic incubations of *S. oneidensis* MR-1 with the replenishment of Fe(III) citrate at 60 d.

Next, since the degradation of O-SWCNTs was expected to require a long period of time (several months or more), we investigated the conditions that would maintain the cell activity of *S. oneidensis* MR-1 and continue the Fenton reaction for a long period of time even in the presence of O-SWCNTs. Theoretically, under anaerobic conditions, this bacterium respirates using lactate as an electron donor and Fe(III) as a final electron acceptor and produces Fe(II). Then, the Fe(II) and H_2_O_2_ produced by the bacterium under aerobic conditions undergo the Fenton reaction, oxidizing Fe(II) to Fe(III) and generating ·OH. Therefore, *S. oneidensis* MR-1 cells were subjected to alternating anaerobic-aerobic incubation in the presence of O-SWCNTs and 10 mM Fe(III) citrate, and the production of Fe(II), H_2_O_2_, and ·OH during the incubation was examined. Up to the first 10 d of the 24-h cycle consisting of 21-h anaerobic and 3-h aerobic incubations, the concentration of Fe(II) increased during the anaerobic period and reached 2.6 ~ 3.9 mM at the end of the 21-h anaerobic period (Figure 2). This result was almost consistent with the results obtained by previous studies in which 4 ~ 6.3 mM Fe(II) was produced after 24 h of anaerobic incubation of *Shewanella* spp. at the same concentration of Fe(III) citrate (Sekar and DiChristina, 2014; Peng et al., 2020). In contrast, Fe(II) became undetectable after each aerobic incubation. After 10 d of the incubation cycle, the amount of Fe(II) produced during the anaerobic period rapidly decreased, and its concentration became lower than 1 mM at 14 d, accompanied by an increase in the pH value of the culture medium. Then, the supplied electron donor was changed from sodium lactate to lactic acid after 20 d of the incubation cycle to return the pH to approximately 7, which restored Fe(II) production after the anaerobic period. Therefore, we performed a long-term incubation of *S. oneidensis* MR-1 for 90 d in the presence of O-SWCNTs and Fe(III) citrate. The pH of the culture medium was maintained at approximately 7 by using sodium lactate when the pH was less than 8 and lactic acid when the pH was above 8 as an electron donor. As a result, Fe(II) production was maintained at approximately 2 mM during the anaerobic period until approximately 60 d, despite an initial decrease from approximately 4 mM and daily fluctuations (Figure 3A). However, after 60 d, the amount of Fe(II) produced gradually decreased, and visible precipitation occurred, even though the pH was controlled near neutral. Then, Fe(III) citrate was added to the culture at 60 d at the same concentration as the initial concentration. This restored Fe(II) production after the anaerobic period to that at the beginning of the incubation cycle, and thereafter, the production of Fe(II) was maintained at a similar level until 90 d (Figure 3B). In the absence of *S. oneidensis* MR-1, no Fe(II) production was consistently observed during the incubation cycle, confirming that Fe(II) is produced by the anaerobic respiration of *S. oneidensis* MR-1. The H_2_O_2_ produced after the aerobic period was approximately 20 μM, a level similar to that reported in previous studies (Peng et al., 2020; Yang et al., 2022), and was maintained over 90 d (Figure 3C). It was also confirmed that H_2_O_2_ was produced by MR-1 cells because it was not detected in the absence of the cells. After each anaerobic-aerobic cycle, the production of ·OH was confirmed, and its concentration tended to change with the concentration of Fe(II) produced after the anaerobic period; ·OH production gradually decreased up to 60 d and was restored completely by the addition of Fe(III) citrate at 60 d (Figure 3D). No ·OH was consistently observed during the incubation cycle in the absence of *S. oneidensis* MR-1, confirming that it is produced through the Fenton reaction by H_2_O_2_ and Fe(II) produced by the bacterium. Thus, important factors were determined, including the nontoxic concentration of O-SWCNTs, pH level, and replenishment of Fe(III) citrate during the incubation cycle, that prolonged the bacterial Fenton reaction in the presence of O-SWCNTs using *S. oneidensis* MR-1 for a long time over 90 d.

### 3.2 Degradation of O-SWCNTs by anaerobic-aerobic incubation with *S. oneidensis* MR-1

**Figure 4:**
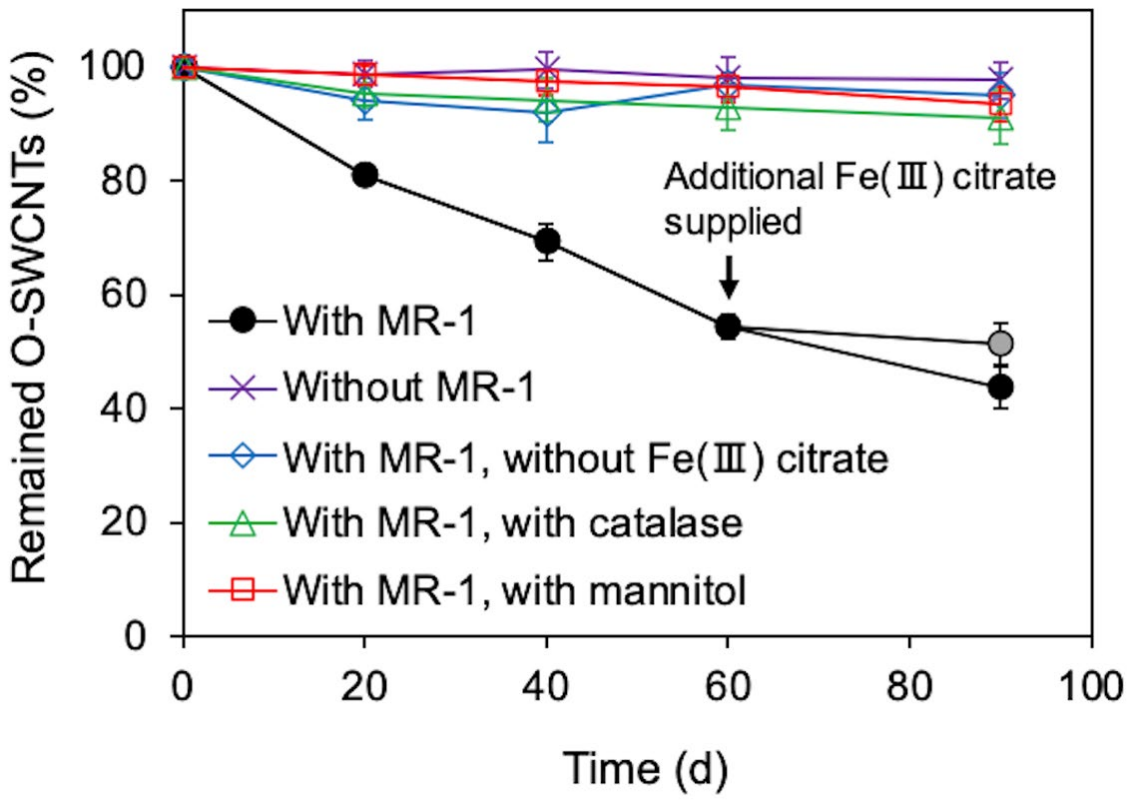
Long-term O-SWCNT degradation via the Fenton reaction driven by *S. oneidensis* MR-1 in 24-h incubation cycles consisting of 21-h anaerobic and 3-h aerobic periods under daily controlled pH near neutral. A gray circular symbol indicates that Fe(III) citrate was not replenished at 60 d. Error bars indicate standard error from three independent incubations.

**Figure 5:**
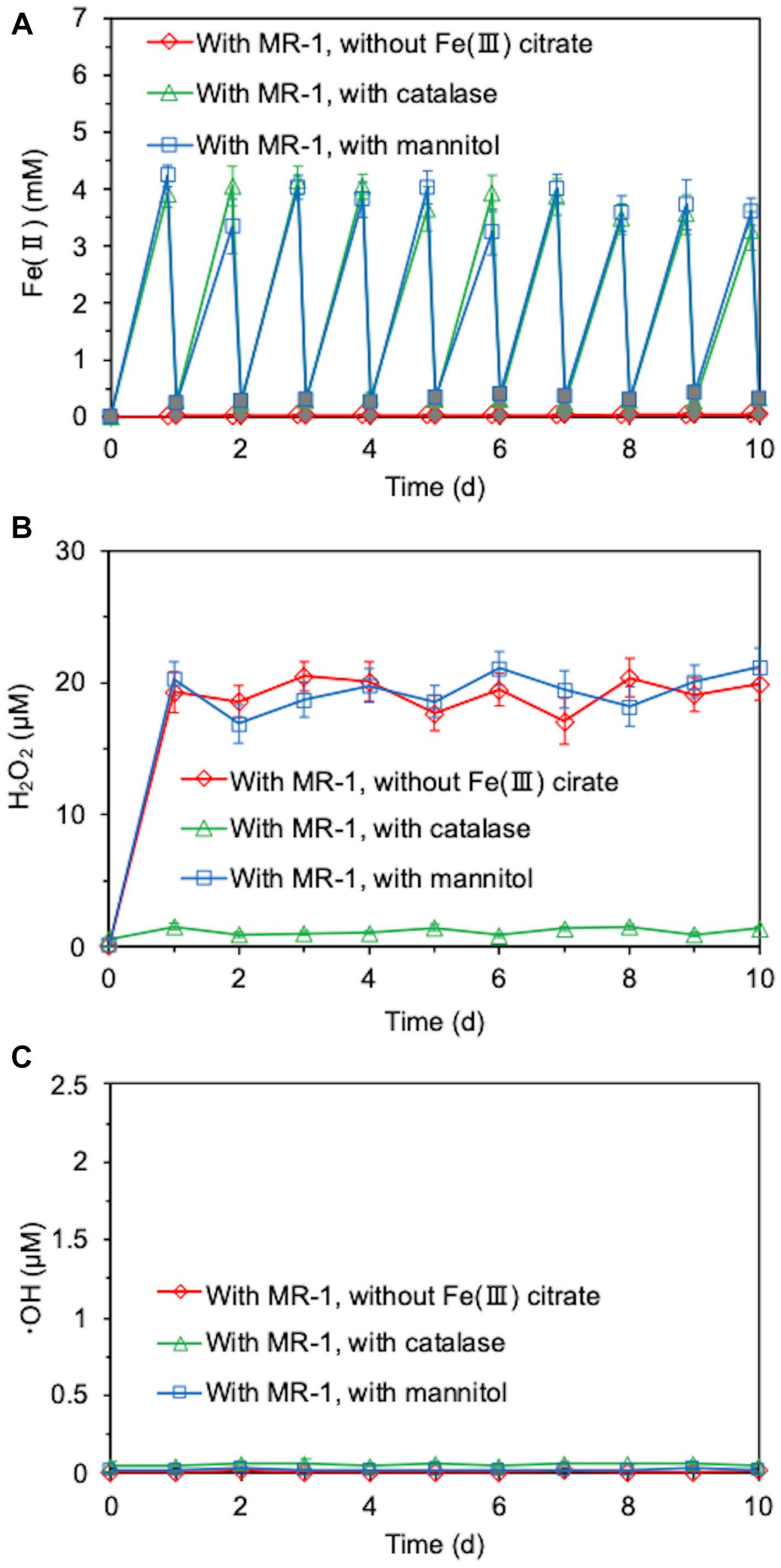
Time courses of Fe(II) **(A)**, H_2_O_2_ **(B)**, and ·OH **(C)** concentrations during the cultivation of *S. oneidensis* MR-1 with 24-h incubation cycles consisting of 21-h anaerobic and 3-h aerobic periods in the presence of O-SWCNTs. Fe(III) citrate was absent from the incubation solution or catalase or mannitol was added to the incubation solution. White and gray symbols in **(A)** indicate Fe(II) concentrations during the anaerobic period and aerobic period, respectively. Error bars indicate standard error from three independent incubations.

We investigated whether long-term continuous degradation of O-SWCNTs is possible by the bacteria-driven Fenton reaction using *S. oneidensis* MR-1 under the conditions described in the previous section. After the bacterial cells and iron precipitate were removed, the concentration of O-SWCNTs during anaerobic-aerobic incubation with *S. oneidensis* MR-1 was quantified by measuring the A_750_ of the O-SWCNT redispersion solution (Yang et al., 2019; Takahashi et al., 2023). When O-SWCNTs were subjected to alternating anaerobic-aerobic incubation with *S. oneidensis* MR-1, at a pH of approximately 7, O-SWCNT degradation progressed continuously with incubation time; however, when Fe(III) citrate was not replenished, the degradation hardly progressed after 60 d, and the degraded fraction remained at 48.7% at 90 d (Figure 4). However, when Fe(III) citrate was replenished at 60 d, O-SWCNT degradation continued thereafter, eventually reaching the degraded fraction of 56.3% at 90 d. No degradation of O-SWCNTs was observed in the control sample without *S. oneidensis* MR-1. To confirm that O-SWCNT degradation was caused by a bacteria-driven Fenton reaction, we prevented the reaction from occurring in the absence of the reactants Fe(II) or H_2_O_2_. Fe(II) was not produced unless Fe(III) citrate was not added to the culture (Figure 5A), but H_2_O_2_ was produced with or without Fe(III) citrate (Figure 5B); the addition of catalase, an enzyme that consumes H_2_O_2_, eliminated H_2_O_2_ (Figure 5B) but did not inhibit Fe(II) production (Figure 5A). The product of the Fenton reaction ·OH did not form in the absence of Fe(III) citrate or in the presence of catalase (Figure 5C). Thus, under these incubation conditions, little degradation of O-SWCNTs occurred (5.0% without Fe(III) citrate and 8.9% with catalase). Furthermore, we investigated the addition of mannitol to the culture, a scavenger of ·OH produced by the Fenton reaction. Mannitol was confirmed to eliminate ·OH (Figure 5C) without any effect on the production of Fe(II) or H_2_O_2_ (Figures 5A,B). In the presence of mannitol, O-SWCNTs were hardly degraded (the degraded fraction was 6.6%). These results suggest that O-SWCNTs were degraded by ·OH generated by the Fenton reaction caused by Fe(II) and H_2_O_2_ produced by the alternating anaerobic-aerobic incubation of *S. oneidensis* MR-1.

### 3.3 Transformation of O-SWCNTs during degradation by *S. oneidensis* MR-1

**Figure 6:**
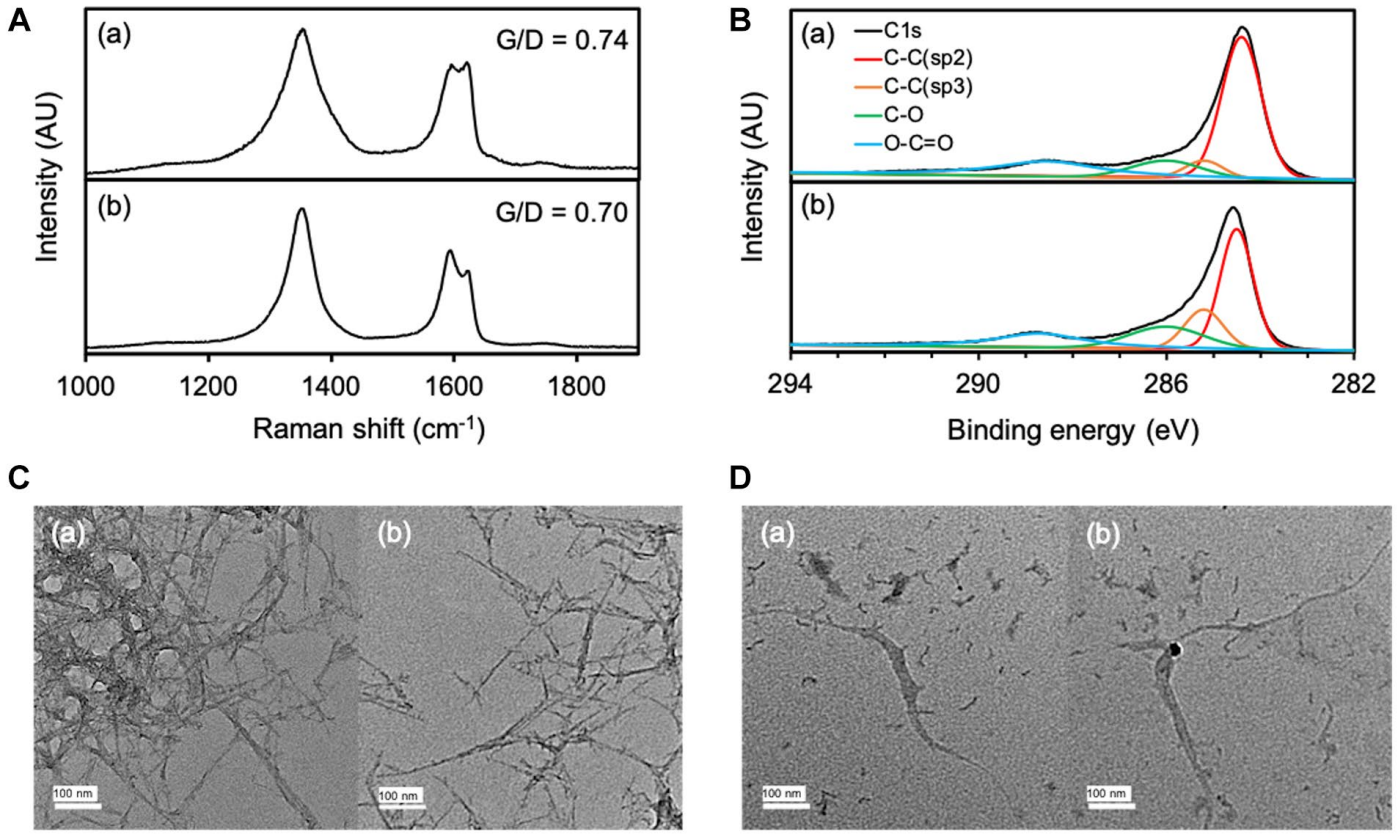
Raman spectra **(A)** and XPS spectra **(B)** of O-SWCNTs at 0 d **(a)** and 90 d **(b)** of incubation with *S. oneidensis* MR-1 in 24-h incubation cycles consisting of 21-h anaerobic and 3-h aerobic periods. TEM images of O-SWCNTs at 0 d **(C)** and 90 d **(D)** of incubation with *S. oneidensis* MR-1 in 24-h incubation cycles consisting of 21-h anaerobic and 3-h aerobic periods. Different views are shown in **(a)** and **(b)**.

The transformation of O-SWCNTs during degradation by the bacteria-driven Fenton reaction using *S. oneidensis* MR-1 was also examined by spectroscopic analyses. The G-band (graphite) peak at 1,591 cm^−1^ and the D-band (disordered) peak at ~1,350 cm^−1^ are characteristic Raman bands of graphitic carbon materials, and the intensity ratio of the G-band to the D-band (G/D) is an index of structural disorder (Figure 6A; Miyata et al., 2011; Flores-Cervantes et al., 2014; Zhang M. F. et al., 2019; Wang et al., 2020). Raman spectra of O-SWCNTs after 90 d of incubation with *S. oneidensis* MR-1 showed a decrease in G/D from the initial value, suggesting an increase in the number of holes and defects on the surfaces of the O-SWCNTs. The trend of Raman spectral change was consistent with previous reports on bacterial biotransformation of CNTs (Chouhan et al., 2016; You et al., 2017; Wang et al., 2020). XPS analysis was performed to further investigate the changes in the chemical composition of O-SWCNTs. The acquired C1s spectrum of the samples resolved into different characteristic peaks (Figure 6B). The peak attributed to sp2 C-C decreased from 57.4 to 44.5% during 90 d of the incubation cycle with *S. oneidensis* MR-1, while the peak attributed to sp3 C-C increased from 6.8 to 16.7%. In addition, the oxygen functional groups (C-O and O-C=O) increased from 35.8 to 38.7%. These results imply that the graphitic structure in O-SWCNTs was oxidized, supporting the above interpretation obtained from the Raman spectral change.

Morphological changes in O-SWCNTs were also confirmed by TEM observation. Before incubation (0 d), O-SWCNTs aggregated with entangled long fibers, and single O-SWCNT fibers were barely visible in any of the fields of view (Figure 6C). In contrast, after 90 d of the incubation cycle with *S. oneidensis* MR-1, most of the O-SWCNT fibers were observed to be short and isolated, along with fiber debris that appeared shredded (Figure 6D). The isolation of O-SWCNTs was presumably due to the progress of oxidation, which reduced van der Waals interactions between the nanotubes (Kim et al., 2012), which is consistent with the results obtained by Raman spectroscopy and XPS analysis.

## 4 Discussion

This is the first report that quantitatively demonstrates continuous bacterial degradation of CNTs for a long period of time over 90 d. We succeeded in O-SWCNT degradation utilizing the Fenton reaction driven by an alternating anaerobic-aerobic incubation cycle of *S. oneidensis* MR-1. Table 1 summarizes methods and results in previous papers about bacterial degradation of CNTs in comparison with those in this study. In all of the previous studies (Zhang et al., 2013; Chouhan et al., 2016; You et al., 2017; Wang et al., 2020, 2021), the degradation of CNTs was evaluated at only one time point; thus, the degradation rates or long-term stability and continuity of the degradation reaction could not be discussed. In addition, in most of the previous studies, the molecular mechanism underlying bacterial degradation of CNTs was unclear. In the studies by Wang et al. (2020, 2021) using *Labry* sp. WJW, CNT degradation was due to a bacteria-driven Fenton reaction, as in our present study. However, in their studies, unlike our present study, the Fenton reaction was induced by Fe(II) produced by the siderophore-mediated reduction of Fe(III). They observed the degraded fraction only after 20-d incubation of CNTs with the bacterium. Therefore, we do not know whether CNT degradation by the siderophore-mediated Fenton reaction can be maintained for a long period of time. The present study showed that the bacteria-driven Fenton reaction can stop in the middle of the process. Degradation of organic pollutants other than CNTs using the Fenton reaction driven by *Shewanella* spp. has also been reported, but the continuation of the Fenton reaction was only monitored for up to 7 d (McKinzi and Dichristina, 1999; Sekar and DiChristina, 2014; Yan et al., 2016; Peng et al., 2020; Yang et al., 2022). We found that to achieve long-term continuous Fenton reaction driven by *S. oneidensis* MR-1, the pH value of the culture medium must remain near neutral and Fe(III) citrate must be replenished during long-term incubation. We need to be careful not to raise the pH during incubation, which could result from the metabolism of the electron donor lactate (Tang et al., 2006). In addition, OH^-^ produced by the oxidation of Fe(II) and Fe(III) in the Fenton reaction may contribute to the pH increase (Brillas et al., 2009). It has been reported that alkaline conditions are unfavorable for microbial reduction of Fe(III) in the anaerobic respiration of *S. oneidensis* MR-1 (Wang et al., 2018; Zhu et al., 2022). Nevertheless, microbially driven Fenton reactions have been carried out without pH control in previous studies on the degradation of contaminants, with the exception of one study (Yang et al., 2022). Even when the pH was maintained near neutral, the Fe(II) production gradually decreased with the generation of visible precipitation after 60 d of the incubation cycle but recovered by the addition of Fe(III) citrate. This decrease in Fe(II) production could be due to the gradual oxidation and precipitation of iron into a form that was less available to *S. oneidensis* MR-1 for anaerobic respiration. It is well known that bare Fe(III) begins to precipitate above pH 3 in the form of oxyhydroxide, which is called iron sludge (Zhang M. H. et al., 2019). Although chelation of iron with citrate avoids iron sludge formation, its stability in bacterial culture for as long as 60 d in the presence of ·OH has never been reported. Therefore, some of the iron may be released from the decomposed citrate during long-term incubation. It was also important to determine the effect of O-SWCNTs on the growth of *S. oneidensis* MR-1 because, unlike many other contaminants, CNTs are known to possess antimicrobial activity (Kang et al., 2008; Liu et al., 2009; Mocan et al., 2017). At concentrations below 30 μg/mL, O-SWCNTs enhanced the growth of *S. oneidensis* MR-1, whereas at concentrations above 40 μg/mL, they inhibited growth. The reason why low concentrations of O-SWCNTs enhance growth is unknown. However, it has been reported that CNTs can enhance or inhibit bacterial growth depending on their concentration and bacterial species (Rodrigues and Elimelech, 2010; Zhang et al., 2015). By determining the conditions to maintain the bacteria-driven Fenton reaction for a long period of time, we succeeded in degrading O-SWCNTs continuously for 90 d and a final degraded fraction of 56.3%, which is the highest value obtained for bacterial degradation of CNTs (Table 1). Based on these results, the time necessary for the complete degradation of O-SWCNTs is approximately 150 d if their degradation by the Fenton reaction driven by *S. oneidensis* MR-1 linearly continues (Supplementary Figure S2). However, as the degradation of O-SWCNTs progresses and their concentration becomes much lower, the degradation might gradually slow down and take longer to complete, as seen in our previous study (Takahashi et al., 2023).

**Table 1:** Reports on CNT degradation by bacteria.

Bacterial strain	Type of CNTs	Maximum degradation ratio (%)	Time period (d)	Continuity evaluation	Concentration of CNTs (μg/mL)	Quantitative evaluation methods	Mechanisms of degradation	Reference
*Shewanella oneidensis* MR-1	O-SWCNT	56.3	90	○	30	Measurement of absorbance of CNTs dispersion at 750 nm	Fenton reaction driven by alternating anaerobic-aerobic incubation	This study
*Labrys* sp. WJW	SWCNTs	33	20	×	100	Weighting of residual CNTs	Fenton reaction caused by Fe^2+^ produced by siderophores secreted under iron-deficient condition (Fe^3+^: 1.2 μM) and H_2_O_2_	Wang et al. (2020)
	O-SWCNTs	10						
	MWCNTs	21.3						Wang et al. (2021)
*Mycobacterium vanbaalenii* PYR-1	MWCNTs	0.07	3	×	90	Measurement of headspace CO_2_ level during incubation with CNTs	Bacterial co-metabolism (No molecuar mechanism)	You et al. (2017)
	O-MWCNTs	0.55						
*Trabusiella guamensis*	MWCNTs	–	30	×	15	–	–	Chouhan et al. (2016)
*Burholderia kururiensis, Delftia acidovorans, Stenotrophomonas maltophilia*	O-MWCNTs (^14^C-labeled)	6.8	7	×	1	Mesurement of ^14^C radioactivity in CO_2_ released during incubation CNTs	–	Zhang et al. (2013)

The Fenton reaction should be among the most effective and suitable methods for removing organic pollutants. Even recalcitrant chemicals, including CNTs, can be degraded by the Fenton reaction (Li et al., 2017; Brillas, 2022; Takahashi et al., 2023). However, the classical Fenton reaction exhibits several drawbacks, such as continuity, operability, safety, and risk of secondary pollution. Although the Fenton reaction basically involves the oxidation of Fe(II) to Fe(III) by H_2_O_2_ along with ·OH generation and the reduction of Fe(III) to Fe(II) by H_2_O_2_, the rate of the latter reaction is 1/6000 of that of the former reaction (Zhang M. H. et al., 2019). Therefore, Fe(III) accumulates without effective cycling between Fe(II) and Fe(III) and begins to precipitate above pH 3, causing secondary pollution by iron sludge. To avoid this, acidic conditions of pH ≤3 must be used for the classical Fenton reaction. In addition, a continuous supply of H_2_O_2_, which is hazardous to handle, is necessary (Zhang M. H. et al., 2019; Brillas, 2022). In the Fenton reaction driven by *S. oneidensis* MR-1, anaerobic respiration promotes the reduction of Fe(III) to Fe(II), and H_2_O_2_ is self-supplied by this bacterium. Although Fe(III) accumulation causing iron sludge may be completely inevitable during a long-term reaction, shot replenishment of Fe(III) citrate can prolong the Fenton reaction and thereby O-SWCNT degradation. Therefore, this would lead to the establishment of safe, low-cost, and effective treatment technology for CNT waste and pollution in the future. The Fenton reaction driven by *Labrys* sp. WJW reported by Wang should also contribute to the development of another effective CNT treatment, but it requires iron-deficient conditions under which siderophores function (Wang et al., 2020). Furthermore, *Shewanella* spp. can produce Fe(II) from ferrihydrite and goethite, iron minerals that are widely present on the earth’s surface (Peng et al., 2020). Given the ubiquity of *Shewanella* spp. and iron in the environment, it is expected that the Fenton reaction can be driven in a wide range of environments by *Shewanella* spp. and has potential applications for continuous *in situ* bioremediation of CNTs.

## 5 Conclusion

We first successfully demonstrated long-term continuous degradation of O-SWCNTs by a bacteria-driven Fenton reaction over 90 d using *S. oneidensis* MR-1 in alternating anaerobic-aerobic incubation cycles. This long-term Fenton reaction was achieved by maintaining a near neutral pH in the presence of O-SWCNTs at a concentration that did not inhibit bacterial growth and by replenishing Fe(III) citrate during the process (at 60 d). The final O-SWCNT degraded fraction after 90 d of incubation was 56.3%, which is the highest bacterial degradation of CNTs ever reported. *S. oneidensis* MR-1 produces Fe(II) from Fe(III) citrate, a final electron acceptor for anaerobic respiration during the anaerobic period, and ·OH is generated through the Fenton reaction by Fe(II) and H_2_O_2_ produced by MR-1 during the aerobic period. ·OH is responsible for O-SWCNT degradation, which is inhibited by scavengers of H_2_O_2_ and ·OH. Since *Shewanella* spp. and iron are ubiquitous in the environment, a bacteria-driven Fenton reaction can be used to degrade CNTs under a wider range of conditions. In addition, the study contributes to developing novel methods for waste treatment and environmental bioremediation against CNTs.

## Data availability statement

The original contributions presented in the study are included in the article/Supplementary material, further inquiries can be directed to the corresponding author.

## Author contributions

ST: Formal analysis, Investigation, Methodology, Writing – original draft. KH: Conceptualization, Writing – review & editing.
