# Supplementary material for: Long-term continuous degradation of carbon nanotubes by a bacteria-driven Fenton reaction
Source: Front Microbiol. 2023 Nov 30;14:1298323. doi: 10.3389/fmicb.2023.1298323 (PMC10720723; doi:10.3389/fmicb.2023.1298323)
Supplement: Supplementary file 1 [file Data_Sheet_1.docx]

Supplementary Material

**Table S1. Components of M1 medium**

|  | **M1 medium** |  |  | **Vitamin mix** |  |  |
| --- | --- | --- | --- | --- | --- | --- |
|  | **Reagent** | **Concentration** |  | **Reagent** | **mg/L** |  |
|  | Water | Final volume of 1 liter |  | biotin | 2.0 |  |
|  | PIPES | 30 mM |  | folic acid | 2.0 |  |
|  | NaOH | 7.5 mM |  | pyridoxine HCl | 10.0 |  |
|  | NH_4_Cl | 28.04 mM |  | riboflavin | 5.0 |  |
|  | KCl | 1.34 mM |  | thiamine | 5.0 |  |
|  | NaH_2_PO_4_ | 4.35 mM |  | nicotinic acid | 5.0 |  |
|  | Na_2_SO_4_ | 1.5 mM |  | pantothenic acid | 5.0 |  |
|  | Minerals | 3 mL/L |  | B-12 | 0.1 |  |
|  | Vitamins | 3 mL/L |  | p-aminobenzoic acid | 5.0 |  |
|  | Amino Acids | 3 mL/L |  | thioctic acid | 5.0 |  |
|  | Sodium lactate | 20 mM |  |  |  |  |
|  |  |  |  |  |  |  |
|  | **Amino Acid Mix** |  |  | **Mineral Mix** |  |  |
|  | **Reagent** | **g/L** |  | **Reagent** | **g/L** |  |
|  | L-glutamic acid | 2.0 |  | NTA | 1.5000 |  |
|  | L-arginine | 2.0 |  | MgSO_4_ | 3.0000 |  |
|  | D-L- Serine | 2.0 |  | MnSO_4_・H_2_O | 0.5000 |  |
|  |  |  |  | NaCl | 1.0000 |  |
|  |  |  |  | FeSO_4_・7H_2_O | 0.1000 |  |
|  |  |  |  | CaCl_2_・2H_2_O | 0.1000 |  |
|  |  |  |  | CoCl_2_・6H_2_O | 0.1000 |  |
|  |  |  |  | ZnCl_2_ | 0.1300 |  |
|  |  |  |  | CuSO_4_・5H_2_O | 0.0100 |  |
|  |  |  |  | AlK(SO_4_)_2_・12H_2_O | 0.0100 |  |
|  |  |  |  | H_3_BO_3_ | 0.0100 |  |
|  |  |  |  | Na_2_MoO_4_ | 0.0250 |  |
|  |  |  |  | NiCl_2_・6H_2_O | 0.0250 |  |
|  |  |  |  | Na_2_WO_4_・2H_2_O | 0.0250 |  |
|  |  |  |  |  |  |  |

**Figure S1.** Bar graph of CFU data at 24 h in Figure 1. Error bars indicate the SE from three independent incubations. **p* < 0.05 means significantly different from the CFU datum at 0 μg/mL of O-SWCNTs (Student’s t-test).

**Figure S2.** Estimation of the time required for complete degradation of O-SWCNTs by anaerobic-aerobic incubation with *S. oneidensis* MR-1 from experimental data. The circles indicate same experimental data as in Figure 4, and the dotted line indicates an approximate straight line.
